# Supplementary material for: Quantitative Proteomic Analysis of Plasma after Remote Ischemic Conditioning in a Rhesus Monkey Ischemic Stroke Model
Source: Biomolecules. 2021 Aug 6;11(8):1164. doi: 10.3390/biom11081164 (PMC8393806; doi:10.3390/biom11081164)
Supplement: Supplementary file 1 [file biomolecules-11-01164-s001.zip › biomolecules-1299813-supplementary.pdf]

Table S1 Differentially expressed proteins in response to the RIC stimulus demonstrating up regulation and down regulation at one week post-RIC.

**Up regulation (n = 3)**	**Down regulation (n = 2)**
Apolipoprotein A-II	Gamma-glutamyl hydrolase precursor
Collectin-10	Protein HEG homolog 1
Mannose-binding protein C isoform X2	

Table S2 Differentially expressed proteins in response to the RIC stimulus demonstrating up regulation and down regulation at two weeks post-RIC.

Up regulation (n = 1)	Down regulation (n = 4)
Glucose-regulated protein precursor	Putative serpin A13
	Protein-lysine 6-oxidase
	Dermcidin
	Phosphatidylcholine-sterol acyltransferase isoform X2

Table S3. Differentially expressed proteins in response to the RIC stimulus up demonstrating regulation and down regulation at three weeks post-RIC.

Up regulation (n = 9)	Down regulation (n = 6)
Alpha-2-macroglobulin isoform X2	Complement factor H-related protein 2 isoform X2
Complement C3	Voltage-dependent calcium channel subunit alpha-2/delta-1 isoform X7
Histone cluster 1, H2aj	Insulin-like growth factor I isoform X3
Procollagen C-endopeptidase enhancer 1PP	Multiple epidermal growth factor-like domains protein 8 isoform X2
T-complex protein 1 subunit gamma	Basement membrane-specific heparan sulfate proteoglycan core protein isoform X2
C4b-binding protein beta chain isoform X2	Putative serpin A13
Platelet glycoprotein Ib alpha chain	
Tubulin beta-4B chain isoform X1	
Histone H3.1t-like	

Table S4. Differentially expressed proteins in response to the RIC stimulus up demonstrating regulation and down regulation at four weeks post-RIC.

Up regulation (n = 9)	Down regulation (n = 2)
Factor IX precursor	Gelsolin isoform X4
Serum amyloid A-4 protein isoform X2	Fibrinogen alpha chain
Apolipoprotein A-II	
N-sulphoglucosamine sulphohydrolase isoform X1	
Complement C1s subcomponent	
Complement C3	
Alpha-2-macroglobulin isoform X2	
Basement membrane-specific heparan sulfate proteoglycan core protein isoform X2	
Apolipoprotein C-II isoform X1	

Table S5. Differentially expressed proteins in response to the RIC stimulus up demonstrating regulation and down regulation at five weeks post-RIC.

Up regulation (n = 6)	Down regulation (n = 8)
Leukocyte elastase inhibitor isoform X1	Phosphatidylcholine-sterol acyltransferase isoform X2
Histone cluster 1, H2aj	Multiple epidermal growth factor-like domains protein 8 isoform X2
Cell surface glycoprotein MUC18	Complement factor H-related protein 2 isoform X2
Tubulin beta-4B chain isoform X1	Putative serpin A13
Histone H3.1t-like	Voltage-dependent calcium channel subunit alpha-2/delta-1 isoform X7
Microtubule-associated protein RP/EB family member 2 isoform X4	L-lactate dehydrogenase B chain isoform LDHB
	Insulin-like growth factor I isoform X3
	Prostaglandin-H2 D-isomerase precursor

Table S6. GO analysis of differentially expressed proteins between baseline and post-one-week RIC samples.

GO classification 1	GO classification 2	Up	Down
Total protein		8	5
Biological Process	Positive regulation of biological process	2	1
	Immune system process	1	0
	Signaling	1	2
	Locomotion	0	1
	**Metabolic process**	3	1
	**Biological regulation**	3	3
	Interspecies interaction between organisms	1	0
	**Cellular process**	4	3
	Developmental process	1	2
	Localization	2	2
	Response to stimulus	1	2
	Multicellular organismal process	1	3
	Negative regulation of biological process	1	2
Cellular Component	Intracellular	4	2
	**Cellular anatomical entity**	8	4
	Protein-containing complex	4	1
Molecular Function	**Binding**	7	3
	Molecular function regulator	3	2
	Catalytic activity	1	0

Table S7. GO analysis of differentially expressed proteins between baseline and post-two-week RIC samples.

GO classification 1	GO classification 2	Up	Down
Total protein		1	4
Biological Process	Developmental process	1	0
	Multicellular organismal process	1	0
	Regulation of biological process	1	1
	Response to stimulus	1	0
	Biological regulation	1	1
	**Cellular process**	**1**	**2**
	Signaling	1	0
	Interspecies interaction between Organisms	0	1
	**Metabolic process**	**1**	**2**
	Positive regulation of biological process	1	0
	Localization	1	0
	Locomotion	1	0
	Negative regulation of biological process	1	1
Cellular Component	**Cellular anatomical entity**	**1**	**2**
	Intracellular	1	0
	Protein-containing complex	1	0
Molecular Function	Binding	1	1
	Molecular function regulator	0	1
	**Catalytic activity**	**1**	**3**

Table S8. GO analysis of differentially expressed proteins between baseline and post-three-week RIC samples.

GO classification 1	GO classification 2	Up	Down
Total protein		8	5
Biological Process	Positive regulation of biological process	2	1
	Immune system process	1	0
	Signaling	1	2
	locomotion	0	1
	**Metabolic process**	**3**	**1**
	**Biological regulation**	**3**	**3**
	Interspecies interaction between Organisms	1	0
	**Cellular process**	**4**	**3**
	Developmental process	1	2
	Localization	2	2
	Response to stimulus	1	2
	Multicellular organismal process	1	3
	Negative regulation of biological process	1	2
Cellular Component	Intracellular	4	2
	**Cellular anatomical entity**	**8**	**4**
	Protein-containing complex	4	1
Molecular Function	**Binding**	**7**	**3**
	Molecular function regulator	3	2
	Catalytic activity	1	0

Table S9. GO analysis of differentially expressed proteins between baseline and post-four-week RIC samples.

GO classification 1	GO classification 2	Up	Down
Total protein		10	2
Biological Process	Developmental process	2	0
	Signaling	1	1
	**Cellular process**	**4**	**2**
	Locomotion	1	0
	**Immune system process**	**4**	**1**
	**Response to stimulus**	**6**	**1**
	**Positive regulation of biological process**	**4**	**2**
	Biological adhesion	0	1
	**Localization**	**4**	**1**
	**Biological regulation**	**5**	**2**
	**Metabolic process**	**5**	**1**
	Negative regulation of biological process	1	2
	Interspecies interaction between organisms	1	1
	**Multicellular organismal process**	**4**	**1**
Cellular Component	Protein-containing complex	4	1
	Intracellular	1	1
	**Cellular anatomical entity**	**9**	**1**
Molecular Function	**Binding**	**9**	**2**
	Catalytic activity	4	0
	Molecular function regulator	5	0

Table S10. GO analysis of differentially expressed proteins between baseline and post-five-week RIC samples.

GO classification 1	GO classification 2	Up	Down
Total protein		6	7
Biological Process	Positive regulation of biological process	1	1
	Metabolic process	1	4
	Locomotion	1	1
	Multicellular organismal process	1	3
	**Cellular process**	**3**	**5**
	Localization	1	2
	**Regulation of biological process**	**2**	**4**
	**Biological regulation**	**2**	**4**
	Negative regulation of biological process	1	3
	Developmental process	0	1
	Signaling	1	2
	Response to stimulus	1	2
Cellular Component	Protein-containing complex	2	1
	Intracellular	4	4
	**Cellular anatomical entity**	**6**	**6**
Molecular Function	Molecular function regulator	1	2
	**Binding**	**4**	**3**
	Catalytic activity	1	3

Table S11. KOG analysis of differentially expressed proteins between baseline and post-one-week RIC samples.

Protein name	KOG id	KOG class definition	Functional categories	Function class definition
Hs4503987	KOG1559	Gamma-glutamyl hydrolase	METABOLISM	Coenzyme transport and metabolism
Hs5453619	KOG4297	C-type lectin	--	--

Table S12. KOG analysis of differentially expressed proteins between baseline and post-two-week RIC samples.

Protein name	KOG id	KOG class definition	Functional categories	Function class definition
Hs16507237	KOG0100	Molecular chaperones GRP78/BiP/KAR2, HSP70 superfamily	CELLULAR PROCESSES AND SIGNALING	Posttranslational modification, protein turnover, chaperones
Hs17453738	KOG2392	Serpin	CELLULAR PROCESSES AND SIGNALING	Defense mechanisms
Hs4557892	KOG2369	Lecithin: cholesterol acyltransferase (LCAT)/Acyl-ceramide synthase	METABOLISM	Lipid transport and metabolism

Table S13. KOG analysis of differentially expressed proteins between baseline and post-three-week RIC samples.

Protein name	KOG id	KOG class definition	Functional categories	Function class definition
Hs4557225	KOG1366	Alpha-macroglobulin	CELLULAR PROCESSES AND SIGNALING	Posttranslational modification, protein turnover, chaperones
Hs10800144	KOG1756	Histone 2A	INFORMATION STORAGE AND PROCESSING	Chromatin structure and dynamics
Hs7427517	KOG3509	Basement membrane-specific heparan sulfate proteoglycan (HSPG) core protein	CELLULAR PROCESSES AND SIGNALING	Posttranslational modification, protein turnover, chaperones
Hs20539011	KOG0364	Chaperonin complex component, TCP-1 gamma subunit (CCT3)	CELLULAR PROCESSES AND SIGNALING	Posttranslational modification, protein turnover, chaperones
Hs4502505	KOG4297	C-type lectin	--	--
Hs4757894	KOG2353	L-type voltage-dependent Ca2+ channel, alpha2/delta subunit	--	--
Hs4504281	KOG1745	Histones H3 and H4	INFORMATION STORAGE AND PROCESSING	Chromatin structure and dynamics
Hs17453738	KOG2392	Serpin	CELLULAR PROCESSES AND SIGNALING	Defense mechanisms
Hs5174735	KOG1375	Beta tubulin	CELLULAR PROCESSES AND SIGNALING	Cytoskeleton
Hs4504071	KOG0619	FOG: Leucine rich repeat	POORLY CHARACTERIZED	General function prediction only
Hs20544382	KOG1388	Attractin and platelet-activating factor acetylhydrolase	--	--

Table S14. KOG analysis of differentially expressed proteins between baseline and post-four-week RIC samples.

Protein name	KOG id	KOG class definition	Functional categories	Function class definition
Hs4557225	KOG1366	Alpha-macroglobulin	CELLULAR PROCESSES AND SIGNALING	Posttranslational modification, protein turnover, chaperones
Hs7427517	KOG3509	Basement membrane-specific heparan sulfate proteoglycan (HSPG) core protein	CELLULAR PROCESSES AND SIGNALING	Posttranslational modification, protein turnover, chaperones
Hs4502495	KOG3627	Trypsin	METABOLISM	Amino acid transport and metabolism
Hs4504165	KOG0443	Actin regulatory proteins (gelsolin/villin family)	CELLULAR PROCESSES AND SIGNALING	Cytoskeleton
Hs4506919	KOG3867	Sulfatase	POORLY CHARACTERIZED	General function prediction only

Table S15. KOG analysis of differentially expressed proteins between baseline and post-five-week RIC samples.

Protein name	KOG id	KOG class definition	Functional categories	Function class definition
Hs10800144	KOG1756	Histone 2A	INFORMATION STORAGE AND PROCESSING	Chromatin structure and dynamics
Hs4757894	KOG2353	L-type voltage-dependent Ca2+ channel, alpha2/delta subunit	--	--
Hs13489087	KOG2392	Serpin	CELLULAR PROCESSES AND SIGNALING	Defense mechanisms
Hs4504281	KOG1745	Histones H3 and H4	INFORMATION STORAGE AND PROCESSING	Chromatin structure and dynamics
Hs5174735	KOG1375	Beta tubulin	CELLULAR PROCESSES AND SIGNALING	Cytoskeleton
Hs10346135	KOG3000	Microtubule-binding protein involved in cell cycle control	--	--
Hs20544382	KOG1388	Attractin and platelet-activating factor acetylhydrolase	--	--
Hs4557892	KOG2369	Lecithin:cholesterol acyltransferase (LCAT)/Acyl-ceramide synthase	METABOLISM	Lipid transport and metabolism
Hs4557032	KOG1495	Lactate dehydrogenase	METABOLISM	Energy production and conversion

Table S16. KEGG analysis of the top 40 differentially enriched pathway between baseline and post-one-week RIC samples.

#Pathway name	Pathway ID
Antifolate resistance	mcc01523
Folate biosynthesis	mcc00790
PPAR signaling pathway	mcc03320
Cholesterol metabolism	mcc04979
Staphylococcus aureus infection	mcc05150
Phagosome	mcc04145
Complement and coagulation cascades	mcc04610

Table S17. KEGG analysis of the top 40 differentially enriched pathway between baseline and post-two-week RIC samples.

#Pathway name	Pathway ID
Glycerophospholipid metabolism	mcc00564
Protein export	mcc03060
Thyroid hormone synthesis	mcc04918
Prion diseases	mcc05020
Cholesterol metabolism	mcc04979
Protein processing in endoplasmic reticulum	mcc04141

Table S18. KEGG analysis of the top 40 differentially enriched pathway between baseline and post-three-week RIC samples.

#Pathway name	Pathway ID
Alcoholism	mcc05034
Systemic lupus erythematosus	mcc05322
Transcriptional misregulation in cancer	mcc05202
Long-term depression	mcc04730
Ovarian steroidogenesis	mcc04913
Dilated cardiomyopathy (DCM)	mcc05414
Aldosterone-regulated sodium reabsorption	mcc04960
Progesterone-mediated oocyte maturation	mcc04914
Hypertrophic cardiomyopathy (HCM)	mcc05410
MAPK signaling pathway	mcc04010
Pertussis	mcc05133
Signaling pathways regulating pluripotency of stem cells	mcc04550
mTOR signaling pathway	mcc04150
Breast cancer	mcc05224
FoxO signaling pathway	mcc04068
Leishmaniasis	mcc05140
Longevity regulating pathway	mcc04211
Proteoglycans in cancer	mcc05205
ECM-receptor interaction	mcc04512
EGFR tyrosine kinase inhibitor resistance	mcc01521
Glioma	mcc05214
Melanoma	mcc05218
p53 signaling pathway	mcc04115
Complement and coagulation cascades	mcc04610
Endocrine resistance	mcc01522
Inflammatory mediator regulation of TRP channels	mcc04750
AMPK signaling pathway	mcc04152
Longevity regulating pathway - multiple species	mcc04213
Phagosome	mcc04145
Gap junction	mcc04540
Prostate cancer	mcc05215
Arrhythmogenic right ventricular cardiomyopathy (ARVC)	mcc05412
Cardiac muscle contraction	mcc04260
Chagas disease (American trypanosomiasis)	mcc05142
Neuroactive ligand-receptor interaction	mcc04080
Oocyte meiosis	mcc04114
Oxytocin signaling pathway	mcc04921
Ras signaling pathway	mcc04014
Hepatitis B	mcc05161
Necroptosis	mcc04217

Table S19. KEGG analysis of the top 40 differentially enriched pathway between baseline and post-four-week RIC samples.

#Pathway_name	Pathway ID
Complement and coagulation cascades	mcc04610
Pertussis	mcc05133
Systemic lupus erythematosus	mcc05322
Staphylococcus aureus infection	mcc05150
Cholesterol metabolism	mcc04979
Fc gamma R-mediated phagocytosis	mcc04666
Leishmaniasis	mcc05140
Viral carcinogenesis	mcc05203
Glycosaminoglycan degradation	mcc00531
PPAR signaling pathway	mcc03320
Chagas disease (American trypanosomiasis)	mcc05142
Neuroactive ligand-receptor interaction	mcc04080
Hepatitis B	mcc05161
Kaposi sarcoma-associated herpesvirus infection	mcc05167
Legionellosis	mcc05134
Herpes simplex virus 1 infection	mcc05168
Lysosome	mcc04142
Tuberculosis	mcc05152
Regulation of actin cytoskeleton	mcc04810
Platelet activation	mcc04611
Proteoglycans in cancer	mcc05205
ECM-receptor interaction	mcc04512
Phagosome	mcc04145
Metabolic pathways	mcc01100

Table S20. KEGG analysis of the top 40 differentially enriched pathway between baseline and post-five-week RIC samples.

#Pathway Name	Pathway ID
Alcoholism	mcc05034
Transcriptional misregulation in cancer	mcc05202
Long-term depression	mcc04730
Ovarian steroidogenesis	mcc04913
Dilated cardiomyopathy (DCM)	mcc05414
HIF-1 signaling pathway	mcc04066
Hypertrophic cardiomyopathy (HCM)	mcc05410
MAPK signaling pathway	mcc04010
Aldosterone-regulated sodium reabsorption	mcc04960
Glycerophospholipid metabolism	mcc00564
Progesterone-mediated oocyte maturation	mcc04914
Signaling pathways regulating pluripotency of stem cells	mcc04550
mTOR signaling pathway	mcc04150
Systemic lupus erythematosus	mcc05322
Arachidonic acid metabolism	mcc00590
Breast cancer	mcc05224
FoxO signaling pathway	mcc04068
Longevity regulating pathway	mcc04211
EGFR tyrosine kinase inhibitor resistance	mcc01521
Glioma	mcc05214
Melanoma	mcc05218
p53 signaling pathway	mcc04115
Cysteine and methionine metabolism	mcc00270
Endocrine resistance	mcc01522
Inflammatory mediator regulation of TRP channels	mcc04750
Propanoate metabolism	mcc00640
AMPK signaling pathway	mcc04152
Longevity regulating pathway - multiple species	mcc04213
Gap junction	mcc04540
Prostate cancer	mcc05215
Arrhythmogenic right ventricular cardiomyopathy (A C)	mcc05412
Cardiac muscle contraction	mcc04260
Oocyte meiosis	mcc04114
Oxytocin signaling pathway	mcc04921
Pyruvate metabolism	mcc00620
Ras signaling pathway	mcc04014
Glucagon signaling pathway	mcc04922
Necroptosis	mcc04217
Adrenergic signaling in cardiomyocytes	mcc04261
Central carbon metabolism in cancer	mcc05230
